# Caveats and Pitfalls of SOX1 Autoantibody Testing With a Commercial Line Blot Assay in Paraneoplastic Neurological Investigations

**DOI:** 10.3389/fimmu.2019.00769

**Published:** 2019-04-12

**Authors:** Raquel Ruiz-García, Eugenia Martínez-Hernández, Milagros García-Ormaechea, Marta Español-Rego, Lidia Sabater, Luis Querol, Isabel Illa, Josep Dalmau, Francesc Graus

**Affiliations:** ^1^Immunology Department, Centre Diagnóstic Biomédic, Hospital Clínic, Barcelona, Spain; ^2^Neuroimmunology Program, Institut d'Investigacions Biomédiques August Pi i Sunyer (IDIBAPS), Barcelona, Spain; ^3^Service of Neurology, Hospital Clinic, Barcelona, Spain; ^4^Neuromuscular Disorders Unit, Hospital de la Santa Creu i Sant Pau, Barcelona, Spain; ^5^Centro de Investigaciones Biomédicas en Red en Enfermedades Raras (CIBERER), Valencia, Spain; ^6^Universitat Autónoma de Barcelona, Barcelona, Spain; ^7^Institució Catalana de Recerca i Estudis Avançats (ICREA), Barcelona, Spain; ^8^Department of Neurology, University of Pennsylvania, Philadelphia, PA, United States

**Keywords:** SOX1, autoantibodies, small-cell lung cancer, paraneoplastic neurological syndromes, line blot

## Abstract

SOX1 autoantibodies are considered markers of small cell lung cancer (SCLC) and paraneoplastic neurological syndromes (PNS) and are usually determined by commercial line blot in many clinical services. Recent studies suggested that SOX1 autoantibodies also occur in patients with neuropathies unrelated to SCLC, questioning the value of SOX1 autoantibodies as paraneoplastic biomarkers. Here, we compared the specificity and sensitivity of a commercial line blot (Euroimmun, Lübeck, Germany) with those of an in house cell-based assay (CBA) with HEK293 cells transfected with SOX1. Overall, 210 patients were included in the study, 139 patients with polyneuropathies without SCLC, and 71 with disorders associated with SOX1 autoantibodies detected with the in-house CBA. Forty one of these 71 cases had been referred to our laboratory for onconeuronal antibody assessment and 30/71 were patients with known PNS and SCLC. None of the patients with polyneuropathies had SOX1 autoantibodies by either line blot or CBA (specificity of the immunoblot: 100%; 95%C.I.: 97.8–100). Among the 71 patients with CBA SOX1 autoantibodies, only 53 were positive by line blot (sensitivity: 74.6%; 95%C.I.: 62.9–84.2). Lung cancer was detected in 37/41 (90%; 34 with SCLC) patients referred for onconeuronal antibody assessment and 34 of them also had a PNS. Our study confirms the association of SOX1 autoantibodies with SCLC and PNS. The line blot test misses 25% of the cases; therefore, to minimize the frequency of false negative results we recommend the use of a confirmatory test, such as CBA, in patients suspected to have a SCLC-related PNS.

## Introduction

SOX1 autoantibodies are serological markers of small cell lung cancer (SCLC).They occur in up to 15% of patients with SCLC independently of the presence of a paraneoplastic neurological syndrome (PNS) (1, 2). The frequency of SOX1 autoantibodies increases to 60% in patients with SCLC and paraneoplastic cerebellar degeneration (PCD) or Lambert-Eaton myasthenic syndrome (LEMS) (3, 4). SOX1 was first identified as the antigen recognized by anti-glial nuclear antibodies (AGNA) which were characterized by immunohistochemistry showing a characteristic pattern of reactivity with the nuclei of Bergmann glia cells (5). SOX1 belongs to the group B of the Sry-like high mobility group box family of proteins, which are highly expressed in the developing nervous system and the Bergmann glia of the adult cerebellum, and in SCLCs (6–8).

Unlike other onconeural antibodies, screening by immunohistochemistry is not recommended for the detection of SOX1 autoantibodies because the sensitivity is low compared with that of immunoblot of recombinant SOX1 protein (3). Currently, there are several commercial antibody tests based on line blots of recombinant SOX1 but comparison with other techniques is lacking and their sensitivity and specificity is unknown. Recent studies using immunoblot or ELISA suggested that SOX1 autoantibodies can occur in serum of patients with idiopathic polyneuropathies questioning the value of SOX1 autoantibodies as biomarkers of PNS associated with SCLC (9, 10).

In this study we assessed the specificity and sensitivity of a commercial line blot test for SOX1 autoantibodies in a large series of patients with several types of neuropathies or PNS. In addition, we examined the clinical features of the patients who were referred at our laboratory for the diagnosis of onconeural antibodies and had SOX1 autoantibodies.

## Methods

### Patients

Sera from 210 patients were investigated for the presence of SOX1 autoantibodies by a commercial line blot of recombinant SOX1 and a cell based assay (CBA) of HEK293 cells transfected with SOX1, and the findings were used to calculate the sensitivity and specificity of the commercial line blot test. The cohort included three subgroups of patients: (1) 139 patients with different types of neuropathies [41 had idiopathic sensory neuropathy, 49 chronic inflammatory demyelinating polyneuropathy (CIDP), 18 paraneoplastic neuropathy associated with tumors other than lung, and 31 anti-MAG positive monoclonal gammopathy; this subgroup was included because SOX2 autoantibodies have been described in patients with paraproteinemias (11)]; (2) 30 patients with paraneoplastic LEMS, PCD, or other PNS and SCLC with SOX1 autoantibodies that had been initially tested by an avidin-biotin immunoperoxidase technique on nitrocellulose filters containing purified phage plaques expressing SOX1 (these patients were included in the initial study that reported the characterization and clinical value of SOX1 autoantibodies) (3); and (3) 41 patients referred to our laboratory for analysis of onconeural antibodies and whose serum showed the presence of SOX1 autoantibodies by CBA. The clinical information of these 41 patients was used to assess the association of SOX1 autoantibodies with PNS and SCLC. Serum samples were deposited in the Biobank of the Institut d'Investigacions Biomèdiques August Pi i Sunyer, and Hospital of Sant Pau (Barcelona, Spain). Written informed consent for the storage and use of the samples for research was obtained from patients or representative family members. The study was approved by the ethics committee of the Hospital Clínic.

### Diagnostic Criteria for SOX1 Autoantibodies

In initial studies, SOX1 autoantibodies were detected by an avidin-biotin immunoperoxidase technique on nitrocellulose filters with purified phage plaques expressing SOX1 (3). Samples of 30 patients from the initial study that had been positive with this technique were examined here with a CBA of HEK293 cells transfected with GFP tagged SOX1 (see below). All 30 samples were also found positive with the CBA assay, which we considered the “gold standard” technique for the diagnosis of SOX1 autoantibodies in the present study.

### Cell Based Assay

HEK293 cells transfected with a plasmid containing the human SOX1 sequence tagged with the green fluorescent protein (GFP) sequence (Origene, RG218236) were fixed with 4% paraformaldehyde for 10 min, permeabilized with 0.3% Triton X-100 for 5 min, and subsequently incubated with patients' sera, diluted 1/40, in PBS-1% bovine serum albumin (BSA) overnight at 4°C, and a fluorescent secondary antibody diluted 1/2000 (goat anti-human Alexa Fluor 594 [Jackson ImmunoResearch, PA, USA]) for 1 h at room temperature. Slides were then mounted with Prolong Gold antifade reagent (Invitrogen, Carlsbad, CA, USA) and the reactivity visualized with an Axioscope Zeiss microscope.

### Line Blot

Serum samples were tested by the commercial immunoblot kit EUROLINE Paraneoplastic Neurological Syndromes 12 Ag (DL 1111-1601-7 G; Euroimmun, Lübeck, Germany) following the manufacturers' instructions at serum dilution 1/100. Test strips were scanned and evaluated for band intensity using the EUROLineScan software (Euroimmun Lübeck, Germany). Following the manufacturer's recommendations, band intensity values >10 were considered as SOX1 autoantibody-positive. Results ≤5 were considered negative and weak bands that showed values between 6 and 10 (borderline range) were also evaluated as negative.

## Results

**Figure 1:**
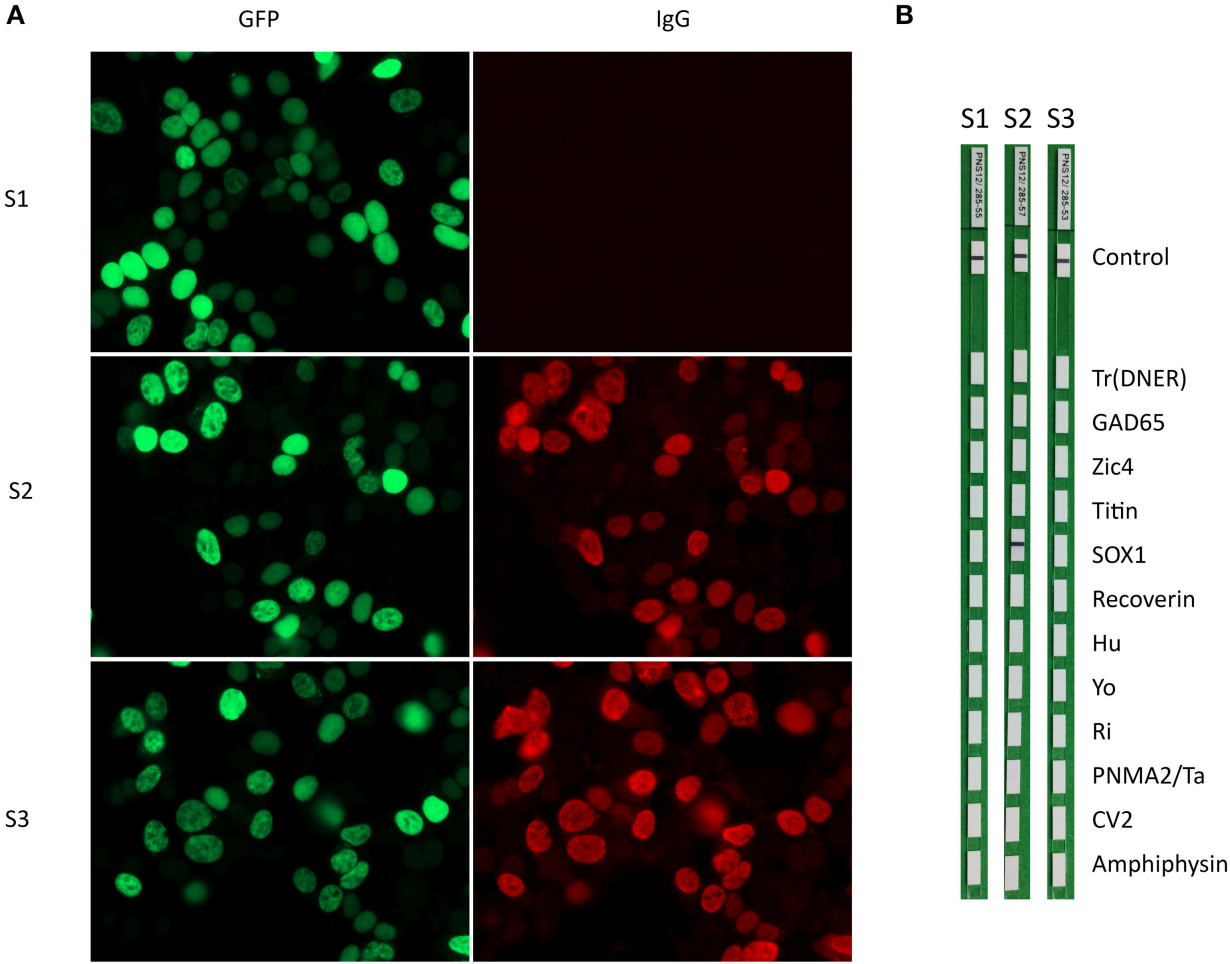
**(A)** HEK293 cells transfected with GFP-SOX1 (green) incubated with (S1) a negative control and two sera positive for SOX1 antibodies (S2 and S3 red). **(B)** The three sera were incubated with strips of the commercial immunoblot (EUROLINE Paraneoplastic Neurological Syndromes 12 antigens). Note that one of the positive sera in the CBA assay is negative by the commercial immunoblot (strip 3).

All 139 sera from patients with polyneuropathies were SOX1 autoantibody-negative by the commercial line blot and SOX1 CBA. The serum of one patient with sensory neuropathy and parotid cancer was positive by immunoblot with a score of 8 (borderline range) provided by the automated band intensity assessment of EUROLineScan software. However, the same serum was negative in CBA of SOX1. In the remaining 71 patients with CBA SOX1 autoantibodies (30 with PNS and SCLC from the initial study and 41 referred to our laboratory for antibody testing) the immunohistochemistry on rat cerebellar sections showed the pattern compatible with AGNA antibodies in 50 of 60 (83%) sera, and the other 11 cases were not assessable due to co-existence of other immunoreactivities. The line blot was positive for SOX1 antibodies in 53 of 71 patients (74.6%) and negative in 18 (24.4%). Ten of the 18 samples showed weak bands of reactivity (borderline range 6–10) and the other eight were negative (≤5) (Figure 1). No clinical differences were noted between these patients and the 18 (25.4%) cases that were SOX1 autoantibody negative by line blot (Table 1).

**Table 1:** Demographic, clinical, and immunological data of 71 patients with SOX1 antibodies according to the commercial immunoblot results.

	**Immunoblot positive *N* = 53 (75%)**	**Immunoblot negative *N* = 18 (25%)**	**P (*t*-student, Chi^**2**^)**
Median age (range)	63 (22–87)	63 (52–74)	0.93
Male/Female (%)	75/25	83/17	0.16
Cancer	52 (98)	17 (94)	0.42
SCLC	49 (94)	15 (88)	0.41
Lung or NSCLC	2 (4)	1 (6)	0.72
Other	1 (2)	1 (6)	0.40
No cancer	1 (2)	1 (6)	0.42
Paraneoplastic syndrome	49 (92)	15 (83)	0.15
PCD	9 (18)	6 (40)	0.08
LEMS	12 (24)	2 (13)	0.36
LE	13 (27)	2 (13)	0.29
Other	15 (31)	5 (34)	0.84
Non-paraneoplastic	4 (8)	3 (17)	0.15
Other antibodies	35 (66)	9 (50)	0.55
AGNA immunoreactivity	38/45 (84)	12/15 (80)	0.69

The specificity of the line blot for diagnosis of SOX1 autoantibodies (proportion of samples without SOX1 autoantibodies that were also negative by the line blot) was 100% (95%C.I.: 97.8–100) and the sensitivity (proportion of samples with SOX1 autoantibodies that were also positive in the line blot) 74.6% (95%C.I.: 62.9–84.2). If we exclude the 30 patients with SOX1 autoantibodies that were selected from our database of PNS, the clinical data of the remaining 41 patients whose samples were sent for onconeuronal antibody testing confirmed the specificity of SOX1 autoantibodies for PNS and lung cancer. Lung cancer was diagnosed in 37 of 41 (90%) patients, 34 [83%] of them SCLC. Only 2 (5%) patients had tumors other than lung cancer (breast, prostate), and no cancer was detected in the other two patients (5%). A PNS was confirmed in 34 of 41 (83%) patients. Among the seven patients without PNS, five had cancer but the cause of the neurological symptoms was metastasis, Wernicke encephalopathy, or non-specific complains, and the other two patients did not have cancer and the cause of neurological symptoms (cerebellar ataxia and fasciculations) was unclear.

## Discussion

The findings of this study confirm the robust association between the occurrence of SOX1 autoantibodies and the presence of lung cancer and show the limitations of the immunohistochemical and line blot assays in the detection of these antibodies. Currently, the preferred screening test for onconeural antibodies in many diagnostic laboratories is the use of commercial line blots which can identify multiple onconeural antibodies in the same strip. The main advantage in using these kits is the simultaneous assessment of multiple onconeural antibodies in a single assay. On the other hand, when these commercial line blots are used as the only antibody screening test, there is an increased risk, which varies for each antibody, of reporting false positive results, downplaying the clinical significance of the autoantibodies. For example, line blot is more sensitive than immunohistochemistry in detecting low titer Hu autoantibodies (12). However, these low Hu antibody titers indicate the presence of a SCLC but do not necessarily confirm that the associated neurological symptoms are paraneoplastic (2, 12). A second limitation of commercial line blots is that in some patients they fail to detect the presence of onconeural antibodies, suggesting they are negative. This is clinically important because the disorder may no longer be considered paraneoplastic and therefore, the search for a tumor felt to be unnecessary. Some autoantibodies seem to be more undetected than others; for example, we previously reported that CV2 (CRMP5) autoantibodies were missed in 7.5% (4/53) of samples that were otherwise anti-CV2-positive by immunohistochemistry or CBA (13). The reason for the negative immunoblot reactivity was unclear but the possibility of very low CV2 antibody titers and the reaction with conformational epitopes were reasonably excluded. In this study, we found the same problem with SOX1 autoantibodies that were undetected in 25.4% of the samples using the commercial line blot. This figure could be reduced to 11.3% if we consider SOX1 autoantibody positive those cases that showed a weak, but visible, immunoblot band (score 6–10 by the EUROLineScan software). However, if this approach is taken there is a risk of false positive results as demonstrated by the weak positive sample in the group of neuropathies that was negative by CBA. A potential solution to increase sensitivity, keeping specificity, would be to test by CBA all samples that show a weak band in the line blot and that are currently considered negative.

We were unable to confirm previous findings of SOX1 antibodies in patients with non-paraneoplastic polyneuropathies (9, 10). A study that investigated the frequency of SOX1autoantibodies in 92 patients with paraneoplastic and non-paraneoplastic neuropathies, found nine positive patients and only 5 (55.5%) of them had cancer (SCLC: four patients; bronchial carcinoid: 1) (9). SOX1 autoantibodies were detected by immunoblot of HEK293 cells transfected with mouse SOX1 clone and the findings were not confirmed with additional techniques (9). Although the homology between human and mouse SOX1 proteins is high we cannot rule out that the discordant results compared with our current findings are due to the use of clones from different species. For example, only 60% of patients with antibodies against human myelin oligodendrocyte glycoprotein (MOG) react with mice MOG (14). In another study, SOX1 autoantibodies were screened by ELISA in a series of 1,493 patients with paraneoplastic and non-paraneoplastic disorders. Fifteen patients (1.0%) showed anti-SOX1 reactivity and nine of them had neuropathies. Only 6/15 patients had cancer, none of them SCLC. However, the presence of SOX1 antibodies was not confirmed with other techniques, a usual requirement in many ELISA assays, and the concentrations of SOX1 antibodies were significantly lower compared with those of controls that had PNS and SCLC, raising doubt on the clinical significance of the findings (10).

We confirmed in the 41 samples with SOX1 autoantibodies that were sent to our laboratory for onconeuronal antibody assessment the robust association with SCLC. The frequency of patients without cancer was only 4.9%, and SCLC was detected in 83% of the patients, figures similar to those seen with anti-Hu autoantibodies (15). In contrast, and compared with other antibodies associated with SCLC and PNS (Hu or CRMP5), the frequency of patients with the final diagnosis of PNS was lower (87%) emphasizing the need to rule out alternative diagnoses for the neurological symptoms that may lead to antibody testing even when the presence of cancer is confirmed.

In summary, findings from this study reveal a diagnostic limitation of a commercial line blot for the detection of SOX1 autoantibodies. A logical approach to circumvent this problem would be the use of a CBA as the primary assay. However, no commercial CBA is available, so in order to minimize the risk of false negative results by line blot, we suggest using in-house CBA testing (available in centers of reference) in all patients whose samples show weak or absent reactivity with line blot but whose clinical syndrome strongly suggest a PNS associated with SCLC (3, 4).

## Ethics Statement

Written informed consent for the storage and use of the samples for research was obtained from patients or representative family members in agreement with the principles of the Declaration of Helsinki. The study was approved by the ethics committee of the Hospital Clínic. The experimental procedure that include animals complies with European and local regulations (2010/63/UE and RD53/2013).

## Author Contributions

FG designed the study and wrote the manuscript in collaboration with JD, RR-G, and EM-H. RR-G, EM-H, MG-O, ME-R, and LS performed laboratory work and/or data analysis. LQ and II send patient samples. All authors discussed the results and commented on the manuscript.

### Conflict of Interest Statement

JD receives royalties from Athena Diagnostics for the use of Ma2 as autoantibody tests and from Euroimmun for the use of NMDAR and GABAB receptor as autoantibody tests and licensing fees from Euroimmun for the use of DPPX, GABAA receptor, and IgLON5 as diagnostic tests. FG receives royalties from Euroimmun for the use of IgLON5 as a diagnostic test. The remaining authors declare that the research was conducted in the absence of any commercial or financial relationships that could be construed as a potential conflict of interest.
